# Food Pattern Modeling as an Alternative Assessment Method to Multiday Dietary Recalls for Iron-Related Nutrients: A Proof-of-Concept Study

**DOI:** 10.3390/nu12071911

**Published:** 2020-06-28

**Authors:** Nicole Delimont, Sarah Nickel

**Affiliations:** 1 School of Nursing, Wichita State University, Wichita KS 67260, USA; 2 Department of Medical Laboratory Sciences, Wichita State University, Wichita, KS 67260, USA; sarah.nickel@wichita.edu

**Keywords:** food pattern modeling, dietary assessment, iron, phytate, ascorbic acid

## Abstract

There are barriers to in-depth memory-based dietary assessment techniques in community-based research. Food pattern modeling may be an alternative method to traditional assessment techniques. The objective of this study was to pilot a comparison of food pattern modeling to 24 h diet recalls for predicting hematological outcomes of iron status. Data from 3–24 h dietary recalls in 27 women were analyzed by two methods: mean dietary intake estimates or food pattern modeling. Food pattern modeling was used to determine the total inventory of foods consumed with iron, phytate, or ascorbic acid or iron–phytate ratios. Each variable was analyzed for its relationship to hemoglobin, ferritin, and acute iron absorption from a meal challenge study by creating receiver operating characteristic (ROC) curves. There were no differences in ROC curves or diagnostic accuracies between food pattern modeling or mean dietary intake estimates for iron, vitamin C, phytate, or phytate–iron ratios for estimating hemoglobin or ferritin values (*p* > 0.05). Food pattern modeling was inferior to mean dietary estimates for acute iron absorption, suggesting that more detailed methods may be necessary for studies with sensitive or acute dietary measurement outcomes. Food pattern modeling for total iron, vitamin C, phytate, and phytate–iron ratios may be comparable to detailed memory-based recalls for larger studies assessing the impact of foods on iron status.

## 1. Introduction

Recently, criticisms of memory-based dietary assessment methods have spurred conversation about the context from which a diet assessment is made. Some dietary assessment methods seek to understand whole food consumption, while others quantify macro- and micronutrients, quantities of phytonutrients, and other active dietary components [1]. While there is real concern about the validity of self-reported dietary intake measurements, there are few truly objective dietary-measurement techniques, necessitating valid and reliable self-reported dietary measurements for nutrition research [1]. One suggestion for researchers interested in specific nutrient adequacy is that the use of a “method that captures frequency of consumption and amounts consumed of all foods and beverages contributing the dietary component of interest with a high degree of validity [1].” The “gold-standard” for this approach would be multiple-pass 24 h diet recalls taken on several days. However, limitations to this method include detail-based memory fault, reporter bias, difficulty obtaining repeated measure recalls, and difficulty obtaining direct histories from patients and participants. These barriers make the process of highly detailed dietary recall assessment methods difficult in some areas of population-based research, particularly in programs that need frequent monitoring for nutrient-based intervention.

Food pattern modeling is an emerging dietary assessment method that has been used for community-based nutritional trials and development of dietary guidelines [2,3,4]. In food pattern modeling, foods consumed are categorized or grouped by their nutrient content and consumption patterns are used to estimate nutrients of interest or dietary adequacy [2]. Unlike food frequency questionnaires or multiple-pass dietary recall methods, food pattern modeling does not give context to the quantity or frequency of food consumption but rather to the foods consumed. Advantages of this dietary assessment method include simplicity, reliability, and cost-effectiveness compared to 24 h diet recalls [3]. Despite this, food pattern modeling has not been rigorously validated against 24 h recall methods to predict nutrient adequacy in clinical trials and its retrospective validity to biochemical or anthropometric measures has not been explored.

While studies have used food pattern modeling to determine dietary iron nutritional adequacy [4,5], there has been no validation of this assessment method against gold standard methods. Adequacy of dietary assessment methods for iron may be particularly important to investigate. Iron is one of the most common micronutrient deficiencies globally [6], and iron deficiency is the target of many local, national, and research-based nutrition intervention programs. Some simple assessment methods for iron intake, such as food frequency questionnaires [7], have been validated. However, food-frequency questionnaires thus far have been hemeprotein focused and may not be applicable to populations consuming diverse or primarily non-heme iron-based diets. Further, it is not well understood whether food patterns rich in phytate [8], dietary iron [9], phytate–iron ratios [10], or ascorbic acid [11], all of which have been linked to regulation of iron bioavailability from food, can be evaluated through food pattern modeling. It is also not known how food pattern modeling for these nutrients compares to more intensive methods of diet recall to accurately predict outcomes related to known hematological markers of iron adequacy, such as hemoglobin, ferritin, or acute iron absorption.

The utility and validity of food pattern modeling depends on first understanding whether it can similarly predict hematological iron-status indices compared to intensive memory-based recall methods. The objective of this study was to compare the diagnostic accuracy of food pattern modeling to 24 h diet recall methods for predicting hematological indices of iron status.

## 2. Materials and Methods

### 2.1. Sample Size

A sample size was calculated using a paired equivalence test for hemoglobin, ferritin, or acute iron absorption differences of <15% between dietary assessment methods and with a correlation of at least 85% between methods using a power of 80% from hematological outcomes among participants on a rolling basis. The greatest of the three sample size calculations was 21 paired tests for ferritin, which was used for analysis.

### 2.2. Study Recruitment, Inclusion Criteria, and Study Preparation

The study protocol for the present study was approved by the Institutional Review Board at Wichita State University (#4396). In brief, fifty-six women responded and were screened by email for inclusion criteria listed below on a rolling basis. Before participation, an informed consent document was signed and all study procedures, risks, and benefits were identified verbally and in writing. Participant inclusion criteria dictated that participants were premenopausal, were aged 18–35 y, were nonobese (BMI ≤ 30.0 kg/m^2^), had no history of oral or gastrointestinal disease, were moderate (≤14 g alcohol per day) or nonalcohol consumers, and were nontobacco users. Participants were excluded if they were pregnant, were breastfeeding, had blood or gastrointestinal disorders affecting iron absorption, or were taking medication that would impair iron bioavailability. Participants were asked to stop iron containing medications and supplements seven days prior to study activities. Of the 56 total participants that were contacted for participation, 30 met inclusion criteria and were enrolled. After study completion, only 27 participants had three completed 24 h dietary recalls needed for study analysis.

### 2.3. Hematological Data Collection

To evaluate dietary recall methods against hematological outcomes, each participant completed an iron-rich meal challenge to assess for iron status and bioavailability. Meal challenges followed a format previously described [12,13]. Prior to the meal, two separate blood samples were collected by venipuncture in a 5-mL serum separator and 3-mL ethylenediaminetetraacetic acid (EDTA) evacuated tubes to measure serum iron (by spectrophotometry), ferritin (by immunoassay, sensitivity 0.1 ng/mL), and complete blood count, including hemoglobin (g/dL) [12,13]. After blood draw, the challenge meal was administered. All meals consisted of a 95-g bagel with 12 g sugar-free strawberry jam (half sprinkled with 18 mg iron as ferrous sulfate), a 90-g banana, and 355 mL of water. Blood samples were drawn to measure acute iron absorption at 210 min post-meal in a 2-timepoint draw to estimate the percentage of maximum iron recovery (% max iron absorption) as described previously [12,13,14]. After study activities were completed, blood and serum were sent to Quest Diagnostics for analysis within 24 h. Serum iron data were used to calculate acute iron absorption for iron bioavailability analysis. Calculations were completed as described previously [12,13,14].

### 2.4. Dietary Analysis

Participants completed three 24 h dietary recalls via the Automated Self-Administered 24-Hour Recall (ASA24) three days prior to the test meal. Dietary data were extracted, and means were calculated for four nutrients of interest related to hematological outcomes: iron, phytate, phytate–iron ratio, and ascorbic acid [15]. To determine the nutrient density for food pattern modeling, variables of interest were assigned to a dichotomous categorical value (either “high” or “low” quantities) according to parameters from previous research [4]. During this process, an electronic spreadsheet (Microsoft Excel) was used to review all individual food items from dietary assessment data for each participant. Based on extracted data, individual food items for dietary recalls were coded into categories based on average nutrient content for each variable of interest per 100 g servings. Using previously defined parameters, the cutoff point between high and low phytate–iron ratios was 1 [16,17]. Food iron and ascorbic acid content were categorized based on iron cutoff values of 0.35 mg/100 g [4,5,18,19,20] and 24 mg/100 g [4,19,20,21,22] respectively. Phytate was categorized using a cutoff point of 50 mg/100 g [19,23,24] (Table 1).

A comprehensive dietary nutrient density profile was created for each participant based on mean variable-nutrient density for all foods consumed. Using categorized food items for each nutrient, a sum score for overall food pattern was tabulated by taking the total number of consumed foods that were categorized with high nutrient density for each variable, divided by the total number of foods consumed.

Food pattern modeling estimated nutrient density (for each variable)

$$=\frac{Total\;number\;of\;nutrient\;dense\;\left(“high”\right)\;foods\;consumedmb\;\;foods\;coumed}{Total\;number\;of\;foods\;consumed\;number\;of\;foods\;consumed}$$

To construct mean dietary intake variables for comparison to food pattern modeling, estimated iron (in milligrams) and ascorbic (milligrams) acid intakes were extracted and means were calculated from three 24 h dietary recalls collected during the study. Food intake logs were downloaded from the Automated Self-Administered 24 h recall for manual calculation of phytate and phytate–iron ratios. During this process, an electronic spreadsheet (Microsoft Excel) was used to review all dietary data for each participant. Food items were referenced from United States Department of Agriculture (USDA) tables and entered in an electronic spreadsheet; phytate amounts were calculated and averaged for each recall [25]. The phytate–iron ratio was calculated using the equation:

$$\frac{Average\;estimated\;phytate\;intake\;\left(mg\right)}{Average\;estimated\;iron\;intake\;\left(mg\right)}$$

To compare mean dietary estimates against food pattern modeling variables for hematological outcomes, the sensitivity, specificity, positive predictive value (PPV), and negative predictive value (NPV) of each method was calculated. Similarity of methods was compared by using a receiver operating characteristic (ROC) curve calculation [26]. The ROC was used to compare continuous hematological variables (hemoglobin, ferritin, and acute iron absorption) against estimated mean nutrient intake and food pattern modeling variables. Curves were created to estimate likelihood ratios for each assessment type by setting discrete values within the continuous hematological data indices to form dichotomous comparisons for analysis.

ROC curves were established using points within the nadir and peak values for hemoglobin, ferritin, and iron absorption among participants. Hemoglobin values ranged from 11.0 to 14.0 g/dL with 0.5 g/dL intervals; ferritin values ranged between 5 and 55 ng/dL with 5 ng/dL intervals, and acute iron absorption values ranged from 0 to 50%, with 5% intervals. ROC parameters were chosen according to participant outcomes. Quartiles for each dietary assessment method variable were calculated to plot ROC curves according to previous studies [4,27], where the values are reported Table 2:

### 2.5. Statistical Analysis

Data were analyzed using SAS statistical software (SAS Studio version 3.8), statistical significance was set at *p* < 0.05, and data are presented as mean ± SD. Before analysis, all data were analyzed for normality and homogeneity of data by Levine’s test of homogeneity.

Fisher’s exact test was used to compare dietary assessment methods against hematological outcomes. From the table analysis, sensitivity, specificity, PPV, and NPV were calculated for each nutrient by each hematological cutoff value. Mean dietary estimates and food pattern modeling method sensitivity and specificity were plotted for ROC curve analysis. The calculated response elements for each dietary analysis method were compared by a Student paired T-test, using *p* < 0.05 as a cutoff for significant differences between methods.

## 3. Results

### 3.1. Demographics and Background

Baseline demographic background is included in Table 3. Participants were aged 19–35 (mean 24.5 ± 4.2 y) and were occasional or moderate alcohol consumers. The mean participant BMI was 23.2 ± 4.2 kg/m^2^. Mean hemoglobin was 13.1 ± 1.0 (range 10.9–14.7 g/dL); ferritin was 30.3 ± 15.1 (range 5–61 ng/dL); and acute iron absorption from the meal study was 14.4 ± 26.0% (range 0–50%).

### 3.2. Dietary Intake

Mean estimated iron and ascorbic acid intakes from the 24 h dietary recall analysis were 14.7 ± 5.9 and 92.9 ± 85.1 mg/d, respectively (Table 4). Fruit and vegetable intake were 1.0 ± 0.8 and 1.8 ± 1.4 cups per day, respectively. The average meat protein intake was 130.4 ± 76.5 g/day, and nut and seed intakes were 19.8 ± 28.3 g/day. The mean phytate–iron ratio was 4.15 ± 1.9, and average dietary phytate intake was 669 ± 547 mg/d. Generally, overall recommendations by the 2015–2020 Dietary Guidelines for Americans (DGA) [28] were met by participants. However, mean iron, folate, and fiber intake were generally low while protein and sugar intake were high compared to age and gender-matched recommendations. Nutrient cutoffs for ROC quartile characteristics are listed in Table 5.

### 3.3. ROC Analysis by Diet Assessment Type for Hemoglobin

**Figure 1:**
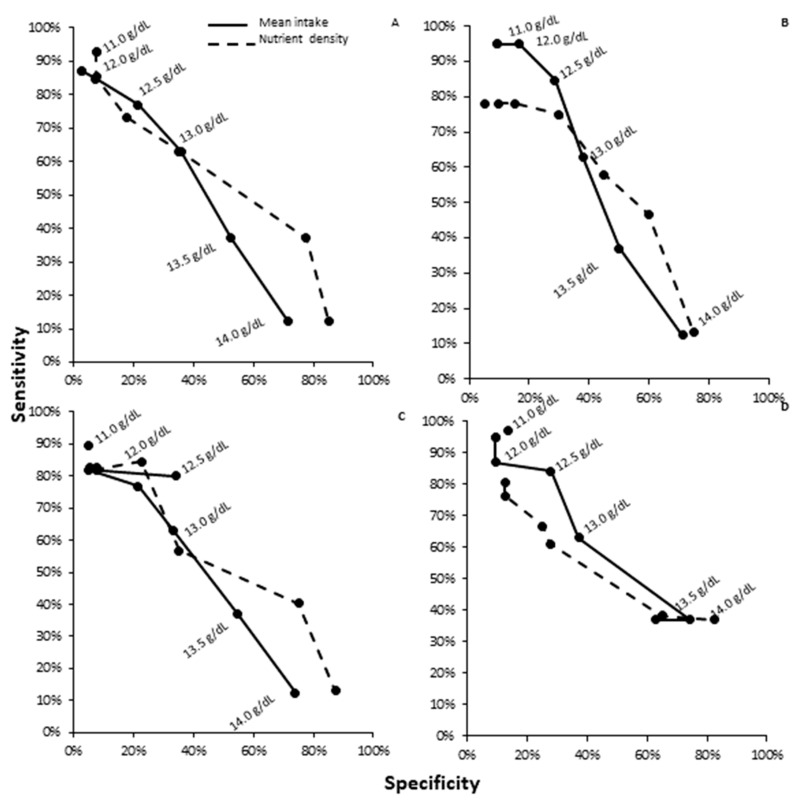
Receiver operative characteristic (ROC) curve for dietary assessment measurement by hemoglobin outcomes: mean intake estimates versus food pattern modeling for (**A**) iron, (**B**) ascorbic acid, (**C**) phytate, and (**D**) phytate–iron ratio for hemoglobin outcomes. There were no significant differences between assessment methods for hemoglobin outcomes prediction (*p* > 0.05).

Overall, food pattern modeling compared to mean nutrient estimates were non-inferior and both were not statistically different for total iron (*p* = 0.7 and 0.06), phytate (*p* = 0.84 and 0.07), vitamin C (*p* = 0.65 and 0.96), or phytate–iron ratios (*p* = 0.15 and 0.06) for hemoglobin outcomes. The ROC curves created for total iron, phytate, vitamin C intake, and phytate–iron ratios indicated that there were no independent differences in likelihood ratios for hemoglobin interval points between analysis methods (Table 6 and Figure 1A–D). The PPVs for iron intake hemoglobin values were 44.3 compared to 51.9% for mean estimated intake or food pattern modeling analysis, respectively, and the NPV values were 44.5 compared to 51.8%, respectively. There were no trends toward superiority for mean estimated intake compared to food pattern modeling analysis methods in predicting hemoglobin values for iron, phytate, vitamin C, or phytate–iron ratios. The mean difference in sensitivity between the two dietary assessment methods was 4.2%, and the mean specificity difference was 4.6%.

### 3.4. ROC Analysis by Diet Assessment Type for Ferritin

**Figure 2:**
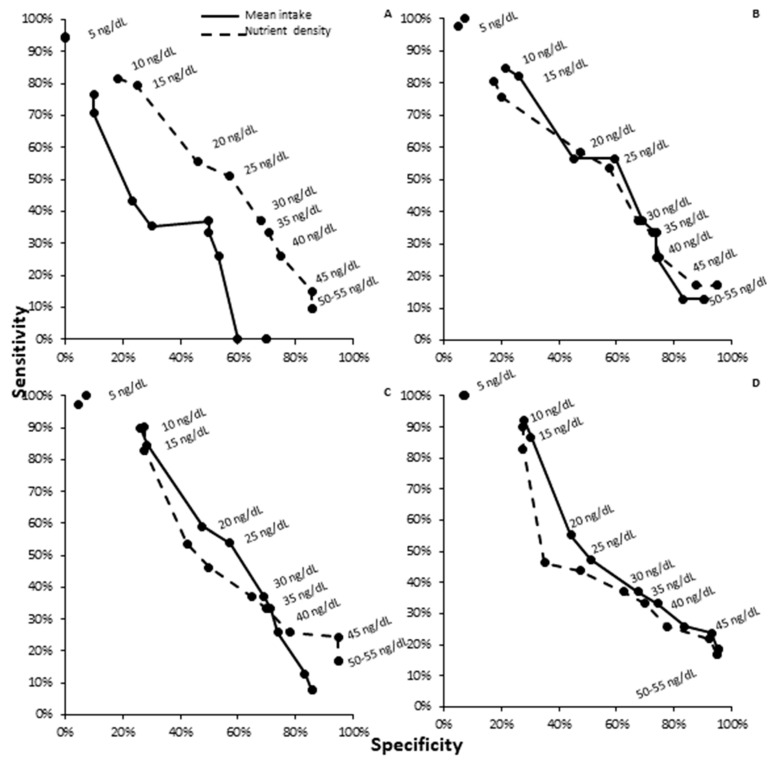
Receiver operative characteristic (ROC) curve for dietary assessment measurement by ferritin outcomes: mean intake estimates versus food pattern modeling for (**A**) iron, (**B**) ascorbic acid, (**C**) phytate, and (**D**) phytate–iron ratio for ferritin outcomes. Nutrient density variables were non-inferior to mean intake estimates for ferritin outcome prediction (*p* > 0.05).

Food pattern modeling compared to mean nutrient estimates was non-inferior and not statistically different for phytate (*p* = 0.09 and 0.19), vitamin C (*p* = 0.37 and 0.39), and phytate–iron ratios (*p* = 0.11 and 0.82) by sensitivity and specificity at any ferritin cutoff value. There was, however, a statistical improvement in diagnostic capacity of food pattern modeling compared to mean dietary estimates for total iron intake related ferritin estimation (sensitivity *p* < 0.0001; specificity *p* = 0.001; Table 6 and Figure 2A–D). There was a mean improvement in sensitivity and specificity for ferritin estimation by food pattern modeling by 17 and 10% respectively compared to mean nutrient estimate analysis, suggesting that food pattern modeling was non-inferior to 24 h recall dietary estimates in predicting ferritin outcomes.

### 3.5. ROC Analysis by Diet Assessment Type for Acute Iron Absorption

**Figure 3:**
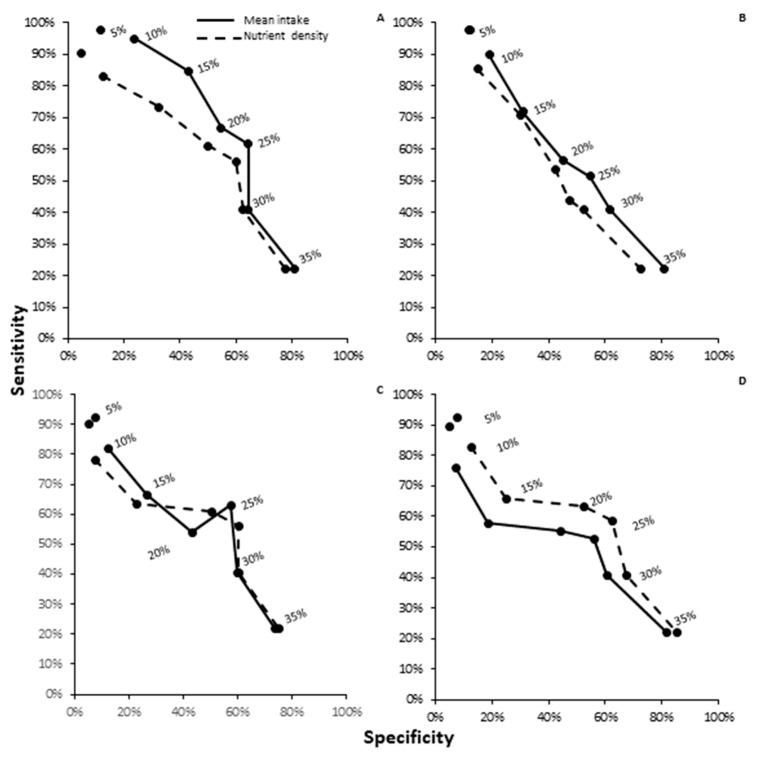
Receiver operative characteristic (ROC) curve for dietary assessment measurement by acute iron absorption outcomes: mean intake versus food pattern modeling for (**A**) iron, (**B**) ascorbic acid, (**C**) phytate, and (**D**) phytate–iron ratio for acute iron absorption outcomes. Nutrient density variables were inferior to mean intake assessments for estimated iron intake and acute iron absorption.

Acute iron absorption estimates were significantly different by dietary assessment method depending on nutrient variable type. For estimated iron intake, the estimated mean intake dietary analysis was superior to food pattern modeling for sensitivity and specificity of iron absorption estimation (42.9 vs. 49.0%, *p* = 0.0012; 61.3 vs. 68.1%, *p* = 0.0063, respectively; Table 6 and Figure 3A–D). Estimated mean vitamin C intake was statistically superior to food pattern modeling for diagnostic accuracy related to acute iron absorption (sensitivity and specificity: 38.9 vs. 43.5%, *p* = 0.008; 57.5 vs. 62.3%, *p* = 0.03, respectively). Despite this, food pattern modeling was non-inferior to mean estimated intake for total phytate (sensitivity: 40.0 vs. 39.8%, *p* = 0.88; specificity: 58.5 vs. 59.7%, *p* = 0.4) and phytate–iron ratios (sensitivity: 44.6 vs. 38.9%, *p* < 0.001; specificity: 63.1 vs. 57.1%, *p* < 0.005).

## 4. Discussion

The objective of this study was to pilot the diagnostic accuracy of food pattern modeling to 24 h diet recall methods. In this study, food pattern modeling was used to predict hematological indices for four variables: iron, ascorbic acid, phytate, and phytate–iron ratios. These variables were chosen due to their known impact on the hematological indices of iron status and to their common focus in community nutrition research and programming [3,4,5,6,7,8,9,10,11,12,13,14,17,18,19,21,22,23,24,27].

Food pattern modeling has been used to estimate community level nutrient intake through modeling methods like the present approach elsewhere [29,30,31,32]. However, this is the first study, to our knowledge, that has compared the effectiveness of food pattern modeling to repeated multiple-pass memory-based dietary assessment by comparing acute and chronic measurements of nutrient status by hematological outcomes. This is also the first study to have compared food pattern modeling and 24 h dietary recall for nutrients with a known relationship to iron status.

Diagnostic measurements, including sensitivity, specificity, PPV, NPV, and ROC curves were not significantly different between food pattern modeling and mean dietary intake estimates for markers of long-term iron status, including hemoglobin and ferritin. Despite this, food pattern modeling was statistically inferior to mean dietary intake estimates for acute iron absorption for estimated iron and ascorbic acid intake. This suggests that, while food pattern modeling may be non-inferior to 24 h dietary recall methods for long-term markers of iron status, it may be inadequate to predict acute markers of iron absorption. However, it has been suggested that dietary iron intake may be poorly predicted by single-meal iron bioavailability studies compared to long-term markers or iron status [3,12,13,33], questioning whether the improved diagnostic capabilities of 24 h dietary recalls may be needed in community-based dietary assessment. In addition, the mean difference in diagnostic accuracy for all nutrient variables for any hematological outcome between assessment methods was less than twenty percent, often trending in favor of food pattern modeling methods for assessment. This suggests of non-inferiority of food-pattern modeling to 24 h recall assessment in this study.

There were several limitations to the generalizability of this study that outline the challenges of food pattern modeling as a dietary assessment method available for widespread use. As a proof-of concept pilot study, hematological outcomes here were correlated with two dietary assessment types approximated from 3–24 h dietary recalls. However, this study was conducted in a homogenous population that largely was not iron deficient. While studying women may be a sensitive group to changes in iron status and absorption, results from this study cannot be generalized to other populations of interest, such as children, pregnant women, or populations with largely anemic cohorts. In addition, this modeling was based on the overall iron intake for hematological outcomes prediction. Future studies may benefit from understanding nuances in nutrient bioavailability during model-building. One such exploration may be to consider differences in modeling between heme and non-heme iron sources. Further, this was a small sample size. Future studies should aim to explore differences in methods using larger sample sizes in populations with geographic diversity.

Despite its definition in the Dietary Guidelines for Americans [2], there is no standardized approach to food pattern modeling as a form of dietary or nutrient assessment. In addition to the methodology from the present study, other studies reviewed suggested a variety of models and methods that were employed with characteristics that met the definition of food pattern modeling [3,4,6,7,8,30,31,32]. To use food pattern modeling broadly, methodology and further validation should be considered for research and programming moving forward.

Finally, this study aimed to model differences in assessment methods for four known nutrient variables that impact iron absorption and status; however, modeling for diverse nutrient, in diverse dietary settings may reveal limitations with food pattern modeling. It is relatively unclear at this time how food pattern modeling can be standardized to populations and programs wishing to utilize this method without complicated modeling efforts. Future studies should outline guidelines and research gaps for researchers and programming intending to use food pattern modeling to estimate nutrient intake for a wide variety of macronutrients and micronutrients.

## 5. Conclusions

Despite limitations, this study was able to validate as a proof-of-concept pilot that food pattern modeling may be as effective as dietary recalls for community health programming or nutrition research. Further, the study was completed in participants consuming a wide diversity of foods when compared to some populations globally. Outcomes generated suggest that food pattern modeling for iron-relevant nutrients was as diagnostically accurate as detailed memory-based recall assessment methods in predicting hematological markers of iron status. This may demonstrate that, with standardized guidelines and further research, food pattern modeling could be a simple, readily utilizable dietary assessment method for research and community health.

## Figures and Tables

**Table 1:** Categorization of nutrient density for food pattern modeling by nutrients of interest.

	High	Low
Phytate–iron ratio (per 100 g)	>1	≤1
Iron content (mg/100 g)	>0.35	≤0.35
Ascorbic acid (mg/100 g)	>24	≤24
Phytate (mg/100 g)	>50	≤50

**Table 2:** Nutrient Quartiles for Receiver Operating Curve Analysis?

Quartile Nutrient Intake	Percentile (%)
Q1	76–100
Q2	51–74
Q3	26–50
Q4	0–25

**Table 3:** Baseline demographics.

Variable	N	Mean
Height (m)	27	1.7 ± 0.1
Weight (kg)	27	64.8 ± 13.3
BMI (kg/m^2^)	27	23.2 ± 4.2
Hemoglobin (g/dL)	27	13.1 ± 1.0
Hematocrit (%)	27	39.4 ± 2.5
MCV (fL)	27	87.9 ± 5.2
MCH (g/L)	27	29.4 ± 2.5
Ferritin (ng/dL)	27	30.3 ± 15.0
C-Reactive Protein (µg/mL)	27	2.8 ± 4.5
Acute iron absorption (%)	27	14.4 ± 26.0

Data are mean ± standard deviation; BMI: Body mass index; MCV: mean corpuscular volume; MCH: mean corpuscular hematocrit.

**Table 4:** Mean nutrient intake from 24 h recall assessment.

Variable	Mean Intake ± SD	Dietary Reference Intake [1]
Number of foods	9.6 ± 2.8	NA
Energy (kcal/day)	1830 ± 473	2000
Protein (g/day)	77.1 ± 22.1	46
Fat (g/day)	73.0 ± 25.4	<78
Carbohydrate (g/day)	209.0 ± 58.2	130
Sugar (g/day)	84.6 ± 35.6	<20
Fiber (g/day)	18.6 ± 6.1	28
Iron (mg/day)	14.7 ± 5.9	18
Zinc (mg/day)	10.2 ± 3.5	8
Copper (mg/day)	1.1 ± 0.4	0.9
Ascorbic acid (mg/day)	92.9 ± 85.1	75
Thiamin (mg/day)	1.7 ± 0.7	1.1
Riboflavin (mg/day)	1.9 ± 0.5	1.1
Niacin (mg/day)	23.7 ± 6.8	14
Pyridoxine (mg/day)	2.0 ± 0.8	1.3
Folate (µg/day)	359.8 ± 111.3	400
Cyanocobalamin (µg/day)	5.9 ± 5.5	2.4
Fruit (g/day)	80.0 ± 64.0	160.0
Vegetable (g/day)	144.0 ± 112	200.0
Starchy vegetable (g/day)	24.0 ± 40.0	55.0
Legumes (g/day)	8.0 ± 16.0	15.0
Grain (g/day)	161.6 ± 68.0	170.0
Meat proteins (g/day)	130.4 ± 76.5	155.0
Soy protein (g/day)	11.3 ± 17.0	8.5
Nuts and seeds (g/day)	19.8 ± 28.3	8.5
Milk (mL/day)	14.8 ± 8.9	700.0
Phytate (mg/day)	669.6 ± 547.4	NA ^i^

^i^ Data are mean ± standard deviation for three 24 h dietary recalls; dietary reference intake for females aged 19–30 according to the 2015–2020 Dietary Guidelines for Americans [1].

**Table 5:** Nutrient cutoffs for receiver operating characteristic (ROC) curves by dietary assessment type.

Dietary Assessment Method	Intake Quartile	Phytate: Iron	Iron	Vitamin C	Phytate
Mean dietary estimates		Ratio	mg	mg	mg
	Q4	<2.9	<9.9	<45	<343.9
	Q3	2.9–4.2	9.9–13.3	45–62.2	343.9–456.7
	Q2	4.2–7.1	13.3–16.3	62.2–99.1	456.7–629.7
	Q1	>7.1	>16.3	>99.1	>629.7
Food pattern modeling		* Nutrient density			
	Q4	<0.35	<0.47	<0.03	<0.11
	Q3	0.35–0.42	0.47–0.55	0.03–0.07	0.11–0.2
	Q2	0.42–0.47	0.55–0.60	0.07–0.14	0.2–0.25
	Q1	>0.47	>0.6	>0.14	>0.25

$*Estimated\;nutrient\;density=\frac{Total\;\#\;\mathrm{high}\;content\;foods\;consumed\;\left(per\;variable\right)}{Total\;\mathrm{high}\;and\;\mathrm{low}\;foods\;consumed\;\left(per\;variable\right)}$.

**Table 6:** Diagnostic accuracy of mean nutrient estimates versus food pattern modeling assessment methods for hematological indices of iron sufficiency.

Outcome	Variable	Method	Sensitivity	Specificity	PPV	NPV	P Sensitivity	P Specificity
Hemoglobin	Iron	MIE	27.6	62.6	44.3	44.5	0.7	0.06
		FPM	33.9	69.3	51.9	51.8		
	Ascorbic acid	MIE	32.0	67.4	51.4	47.9	0.65	0.96
		FPM	36.2	62.4	46.6	51.8		
	Phytate	MIE	32.5	62.4	49.0	45.3	0.84	0.07
		FPM	36.8	62.3	45.5	53.5		
	Phytate: iron ratio	MIE	33.2	70.5	55.2	49.1	0.15	0.06
		FPM	35.5	61.5	44.9	51.9		
Ferritin	Iron	MIE	28.1	50.1	24.9	54.2	<0.0001 *	0.001 *
		FPM	45.4	60.1	39.2	66.0		
	Ascorbic acid	MIE	45.1	61.7	55.7	51.2	0.37	0.39
		FPM	43.9	60.3	51.9	52.4		
	Phytate	MIE	45.9	62.6	57.0	51.8	0.09	0.19
		FPM	45.6	61.9	54.0	53.6		
	Phytate: iron ratio	MIE	43.9	60.3	51.9	52.4	0.11	0.82
		FPM	47.8	65.0	60.8	52.4		
Acute iron absorption	Iron	MIE	49.0	68.1	62.3	55.4	0.0012 *	0.0063 *
		FPM	42.9	61.3	51.9	52.4		
	Ascorbic acid	MIE	43.5	62.3	55.4	50.6	0.008 *	0.03 *
		FPM	38.9	57.5	47.2	49.1		
	Phytate	MIE	39.8	59.7	50.6	48.8	0.88	0.4
		FPM	40.0	58.5	48.5	50.0		
	Phytate: iron ratio	MIE	38.9	57.1	50.6	45.2	<0.001 *	0.005 *
		FPM	44.6	63.1	54.1	53.9		^ii^

^ii^ Outcomes are expressed as percentage. PPV: positive predictive value; NPV: negative predictive value; * *p* < 0.05. Mean intake estimates (MIE): average estimated nutrient intake from 3–24 h dietary recalls on the ASA24:2018 [2]; Food pattern modeling (FPM) according to nutrient density of each variable, where $Estimated\;nutrient\;density=\frac{Total\;\#\;\mathrm{high}\;content\;foods\;consumed\;\left(per\;variable\right)}{Total\;\mathrm{high}\;and\;\mathrm{low}\;foods\;consumed\;\left(per\;variable\right)}$.
